# Attitudes of relatives of patients with schizophrenia

**DOI:** 10.1192/j.eurpsy.2021.1380

**Published:** 2021-08-13

**Authors:** I. Lajmi, S. Omri, M. Maalej Bouali, N. Smaoui, R. Feki, J. Ben Thabet, L. Zouari, N. Charfi, M. Maalej

**Affiliations:** Psychiatry C Department, Hedi chaker University hospital, sfax, Tunisia

**Keywords:** families’ beliefs, schizophrénia

## Abstract

**Introduction:**

Investigating family member’s attitudesabout schizophrenia is an important step in its management. They likely influence the family’s help-seeking decisions and affect both adherence with biomedical interventionsand social integration of the patients.

**Objectives:**

Describe families’ beliefs about the symptoms and the treatments of schizophrenia.

**Methods:**

We led a transversal study including 32 relatives of patients with schizophrenia (DSM 5). We collected data for both patients andrelatives. We asked relatives to respond by “yes/no/I am not certain” to the questionnaireincluding items dealing with symptoms and optimal cures forschizophrenia.

**Results:**

The mean age of the relatives was 60.8 years; 71.9% were parents; 37.5% were illiterate; 46.9% reported having another family member with a mental disorder (MD) and 15.5% of relatives were able to label the term “schizophrenia”. Nine participants (28%) believed that the patient makes shame to the family’s member and 72% of them was convinced that patient is dangerous.The majority of participants (90.6%) provedthe need for drugs and 65.6% attested the utility of psychotherapies.However, they believed innon-medical practices such as reading Holy Koran verses (87.5%), charity and exorcism (62.5%). Family history of MD was correlated to traditional practices (p=0.038). The belief that patient is dangerous and that he makes shame were associated with advanced age of relatives (p=0.000 and 0.037 respectively). Significant correlationwas found betweennon medical practices and erratic follow-up (p=0.043).

**Conclusions:**

This study points outthe need to improve the psychoeducation of family members of persons with schizophrenia.

